# Debinding of Yttria-Stabilised Zirconia/Bimodal Stainless Steel 316L Bi-Materials Produced through Two-Component Micro-Powder Injection Moulding

**DOI:** 10.3390/polym16131831

**Published:** 2024-06-27

**Authors:** Al Basir, Abu Bakar Sulong, Norhamidi Muhamad, Afifah Z. Juri, Nashrah Hani Jamadon, Farhana Mohd Foudzi, Nabilah Afiqah Mohd Radzuan

**Affiliations:** Department of Mechanical and Manufacturing Engineering, Faculty of Engineering and Built Environment, Universiti Kebangsaan Malaysia, Bangi 43600, Selangor, Malaysia; al.basir005@yahoo.com (A.B.); norhamidi@ukm.edu.my (N.M.); afifahjuri@ukm.edu.my (A.Z.J.); nashrahhani@ukm.edu.my (N.H.J.); farhana.foudzi@ukm.edu.my (F.M.F.); afiqah@ukm.edu.my (N.A.M.R.)

**Keywords:** 3YSZ/bimodal SS 316L micro-components, two-component micro-powder injection moulding, debinding, thermogravimetric analysis, sintering

## Abstract

The fabrication of bi-material micro-components via two-component micro-powder injection moulding (2C-µPIM) from 3 mol% yttria-stabilised zirconia (3YSZ) and micro/nano bimodal stainless steel 316L (SS 316L) powders has received insufficient attention. Apart from this, retaining the bonding between ceramic and metal at different processing stages of 2C-µPIM is challenging. This study investigated the solvent and thermal debinding mechanisms of green bi-material micro-parts of 3YSZ and bimodal SS 316L without collapsing the ceramic/metal joining. In this research, feedstocks were prepared by integrating the powders individually with palm stearin and low-density polyethylene binders. The results demonstrated that during the solvent debinding process, the palm stearin removal rate in the bi-materials composed of 3YSZ and bimodally configured SS 316L feedstocks intensified with an increase in temperature. The establishment of interconnected pores in the solvent-debound components facilitated the thermal debinding process, which removed 99% of the binder system. Following sintering, the debound bi-materials exhibited a relative density of 95.3%. According to a study of the microstructures using field emission scanning electron microscopy, an adequate bond between 3YSZ and bimodal SS 316L was established in the micro-part after sintering. The bi-material sintered at 1350 °C had the highest hardness of 1017.4 HV along the joining region.

## 1. Introduction

Powder injection moulding (PIM) is a state-of-the-art manufacturing technology that allows near net-shaped metallic or ceramic components to be mass-produced inexpensively [1,2]. A prospective commercial method for producing micro-sized components is the micro-powder injection moulding (µPIM) technique, which is an adaptation of the PIM process [3,4,5,6]. The manner in which the global market perceives micro-sized components has undergone a profound shift in recent years, with the current outlook being to combine multifarious functional aptitudes into a single micro-component. In response, two-component micro-powder injection moulding (2C-µPIM) was developed as an alternative to µPIM, enabling the micro-scale joining of two different materials to produce bi-material micro-components with several functions [7,8]. The fundamental processing stages of 2C-µPIM are mixing, injection moulding, debinding, and sintering, which are comparable to those of PIM and µPIM. The production of the feedstocks, which includes mixing two distinct categories of powder particles with binders separately, typically commences the 2C-μPIM process. Green bi-material micro-components are fabricated during the injection moulding process using the prepared feedstocks and on the basis of a sequential or simultaneous mechanism. After the debinding of the green bi-materials to eradicate the binders, sintering is carried out with the objective of providing the porous components with a reasonable mechanical strength for use and handling.

According to Rajabi et al. [9], a key criterion of µPIM is that the average particle size of the powders should be at least ten times smaller than the minimum feature size. In accordance with such a viewpoint, the 2C-µPIM process, which is a variation of the µPIM process, necessitates the use of ultra-fine and/or nanopowder particles to fabricate bi-material micro-components. Nanopowders offer a greater potential for usage in 2C-µPIM feedstock as they manifest prominence in surface finish, dimensional stability, densification behaviour, and mechanical properties [10,11,12]. These particles, however, could bring about several difficulties for the 2C-µPIM process. The large specific surface area of nanopowders reduces the loading of powder in the feedstock, which leads to significantly higher shrinkage [13]. Moreover, the reduction in the powder content of the feedstock as a consequence of the large specific surface area increases the viscosity of the feedstock because of the frictional resistance within the particles and makes the injection moulding process exceedingly challenging [11,14]. Introducing a micro/nano bimodal powder system could assist in resolving these constraints in the 2C-µPIM process; such a powder system is typically developed by mixing a micropowder with a nanopowder of the same theoretical density. The drawbacks of the nanopowder are decreased as a result of such mixing, but its advantages are retained [15]. Since the previous two decades, bimodally configured powders have been effectively used in various PIM and µPIM studies to improve the relative density and mechanical properties of the sintered components [9,16,17,18].

In the present work, 3 mol% yttria-stabilised zirconia (3YSZ) and micro/nano bimodal stainless steel 316L (SS 316L) were chosen as the materials used. 3YSZ has a combination of high-temperature stability, good biocompatibility, excellent flexural strength and fracture toughness, and an optimal dielectric constant [19,20]. SS 316L is a prominent metal with a variety of useful features, including decent corrosion resistance, excellent mechanical strength, and ease of production [21,22,23]. Biomedical devices, including dental prosthesis, orthopaedic implants, and surgical instruments, can considerably benefit from the micro-level joining of 3YSZ and SS 316L. Hanemann et al. [24] studied the feedstock properties used to produce zirconia micro-components through μPIM. They reported that the feedstock containing stearic acid with a concentration of 2.2 mg/m^2^ exhibited the best flow behaviour and homogeneity. Foudzi et al. [25] produced μPIM-processed micro-sized zirconia components with a hardness and flexural strength of 900 HV and 400 MPa, respectively. Liu et al. [26] conducted an investigation on the mouldability and sinterability of zirconia micro-gears, revealing that the μPIM technique was successfully used to fabricate micro-gears of 200 μm. In contrast, Oh et al. [17] produced PIM-processed SS 316L components by using micro/nano bimodal SS 316L powders. They reported that the addition of 25 vol.% nanoparticle content to the micropowder increased the relative density of the sintered parts from 98.3% to 99.6%. In the same vein, Rajabi et al. [9] fabricated SS 316L micro-sized components on the grounds of µPIM by employing bimodally configured SS 316L powders and reported that the hardness value of sintered samples increased from 182 HV to 221 HV due to the addition of 30 wt.% nano-sized powder to the micro-sized powder. While studies have been conducted to produce zirconia and micro/nano bimodal SS 316L components through the PIM and µPIM techniques, reports outlining the characteristics of feedstocks, mouldability, debinding behaviour, and sintering for fabricating the bi-material micro-sized components of zirconia and bimodal SS 316L using 2C-µPIM are profoundly inadequate.

In view of the above, in the present study, green bi-material micro-components of 3YSZ and micro/nano bimodal SS 316L were fabricated successfully through 2C-µPIM, and the processes implemented to remove the binders from these components were addressed in order to produce bi-materials devoid of defects during the sintering stage.

## 2. Materials and Methods

### 2.1. Materials

**Figure 1:**
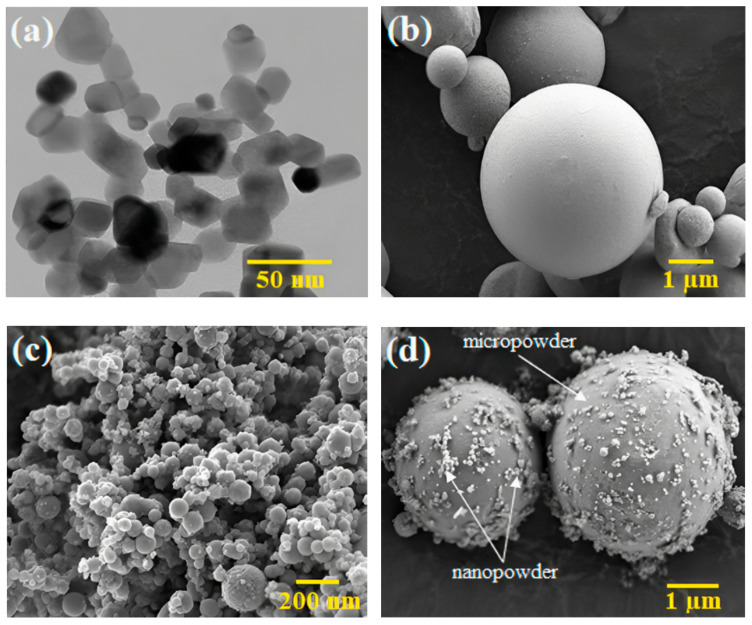
Morphology of the powders: (**a**) TEM image of 3YSZ powder, (**b**) FESEM image of micro-sized SS 316L powder, (**c**) FESEM image of nano-sized SS 316L powder, and (**d**) FESEM image of bimodal SS 316L powder.

**Figure 2:**
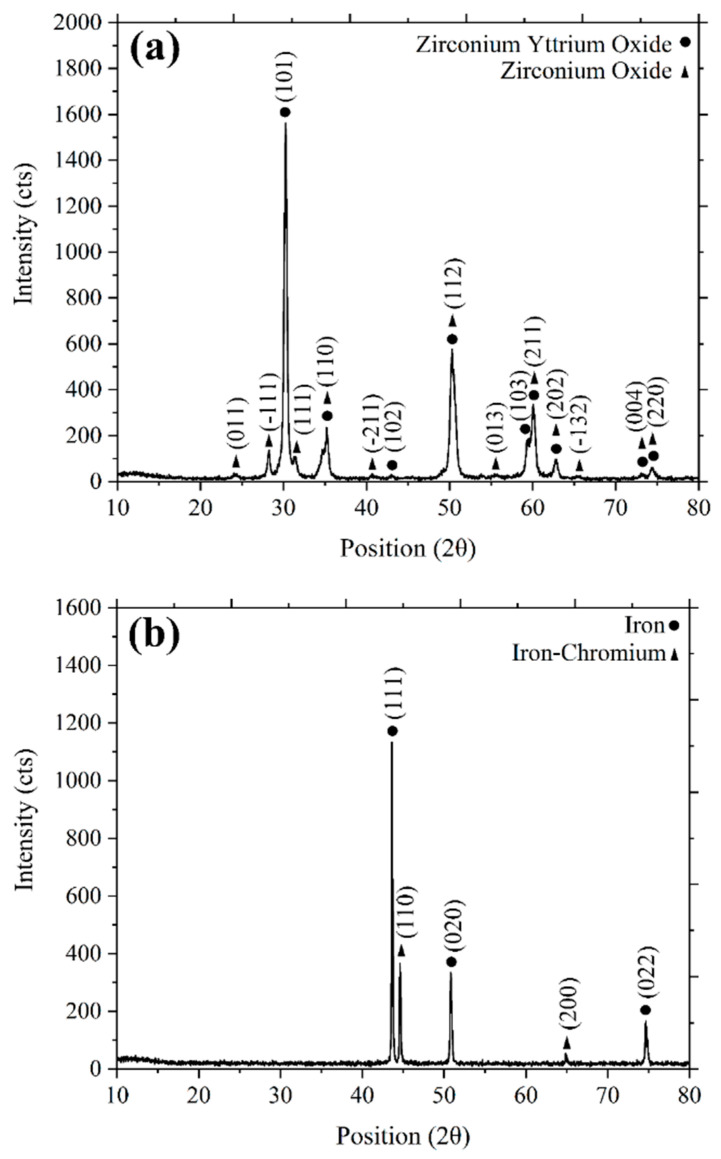
XRD patterns of (**a**) 3YSZ powder and (**b**) bimodal SS 316L powder.

3YSZ and SS 316L powders were used for this research work. The 3YSZ powder had an average particle size of 30 nm. The micro/nano bimodal SS 316L powder was prepared by blending SS 316L powders with average particle sizes of 7 µm and 150 nm, respectively, using a Fritsch Pulverisette-6 planetary mono mill for a time period of 3 h at a rotational speed of 100 rpm. The nanoparticle content in the micro/nano bimodal SS 316L powder was 10 vol.%. The average particle size of the bimodal SS 316L powder was 6.4 µm. The particle sizes were measured using a Malvern Mastersizer 2000 particle size analyser. Pycnometer densities for 3YSZ and bimodal SS 316L were measured using the AccuPyc II 1340 Gas Displacement Pycnometry System, yielding values of 6.0308 g/cm^3^ and 7.7189 g/cm^3^, respectively. The morphology of the powders (Figure 1a–d) was observed using a transmission electron microscope (TEM, Talos L120C) and a field emission scanning electron microscope (FESEM, Zeiss Merlin Compact). The phase analysis of the 3YSZ and bimodal SS 316L powders was examined using X-ray diffraction (XRD, Bruker D-8 advance). The XRD pattern of the 3YSZ powder presented in Figure 2a depicts sharp peaks at 2θ= 30°, 35°, 43°, 50°, 59°, 60°, 62, 73°, and 74°, which are indicative of the existence of a crystalline tetragonal structure of zirconium yttrium oxide according to the crystallography open database (COD, 01-083-0113). The presence of a crystalline monoclinic structure of zirconium oxide is also represented by the peaks at 2θ= 24°, 28°, 31°, 35°, 40°, 50°, 55°, 59°, 62°, 65°, 73°, and 74°, in accordance with COD (01-078-0047). In contrast, Figure 2b illustrates the XRD pattern of the bimodal SS 316L powder. In this figure, the sharp peaks at 2θ= 43°, 50°, and 74° are representative of a face-centre cubic structure of iron, based on the COD (96-901-4712). In addition, the peaks at 2θ= 44° and 64° indicate the presence of the crystalline cubic phase of the iron–chromium composition, as per COD (00-034-0396).

The binder system in the current study was designed to contain 60 wt.% palm stearin and 40 wt.% low-density polyethylene (LDPE). Palm stearin was a primary component of the binder system, which ameliorated its flow properties and wettability. LDPE served as the backbone polymer, strengthening the green components [27]. The melting point of the binders used in the feedstocks was ascertained by a differential scanning calorimetry (DSC) analysis performed using NETZSCH DSC 214 Polyma. Using the NETZSCH Simultaneous Thermal Analyser (STA) 449 F3 Jupiter, a thermogravimetric (TGA) analysis was conducted to estimate the degradation temperature of the binders. In a nitrogen environment, both DSC and TGA were carried out at a heating rate of 10 °C/min. The DSC and TGA data were used as reference points for assessing the temperature during the mixing and debinding procedures [28,29,30].

### 2.2. Feedstock Preparation and Flowability Analysis

In this work, both the 3YSZ and bimodal SS 316L powders had their critical powder volume concentration (CPVC) determined using the oil absorption technique [31]. With a Brabender mixer (W50 EHT), with a temperature setting of 150 °C and a steady blade rotation speed of 30 rpm, the feedstocks of 3YSZ and bimodal SS 316L were produced by autonomously mixing 44 vol% of 3YSZ and 69 vol% of bimodal SS 316L with the binders. Both feedstocks were prepared in the mixer for a mixing duration of 1 h. DSC and TGA were used to analyse the thermal properties of the fabricated feedstocks. The viscosity of the 3YSZ and bimodal SS 316L feedstocks was measured at 130 °C, 180 °C, and 230 °C by using a Shimadzu CFT-500D capillary rheometer. These data helped forecast how the feedstocks would flow as they filled the mould cavity.

### 2.3. Micro-Injection Moulding Process (Green Part Preparation)

With the prepared feedstocks, green bi-material micro-parts of 3YSZ and micro/nano bimodal SS 316L were fabricated using a DSM Xplore plunger-type semi-automatic injection moulding machine. Micro-samples in the shape of dumbbells, measuring 9 mm in length, 2.82 mm in diameter, and 0.9 mm in thickness, were produced using the mould cavity. The parameters used to produce the green components are listed in Table 1. Green 3YSZ/bimodal SS 316L micro-parts in this experiment were fabricated using a sequential mechanism [32]. This method involved initially injecting the 3YSZ feedstock into the mould cavity. Subsequently, the green 3YSZ micro-part was retrieved, split in half, and reinserted into the mould cavity. Finally, the injection of the bimodal SS 316L feedstock was performed over the 3YSZ feedstock to form a bond. Demoulding was conducted with meticulousness following the injection and sufficient cooling of the mould to prevent sample deformation.

### 2.4. Debinding Process (Brown Part Preparation)

In this investigation, solvent and thermal debinding were used to remove the binder, producing the brown pieces. The green 3YSZ/bimodal SS 316L micro-parts were subjected to 40 min of solvent debinding by being submerged in acetone at varying temperatures between 40 °C and 70 °C in a BINDER FDL 115 safety drying oven. Observing the mass loss for five samples under the same processing conditions allowed for the evaluation of the palm stearin removal rate. The single material 3YSZ and bimodal SS 316L micro-specimens solvent debound at 70 °C were subjected to TGA in order to comprehensively understand the context of palm stearin removal from the 3YSZ and bimodal SS 316L areas of the 3YSZ/bimodal SS 316L micro-specimen. The leftover palm stearin after solvent debinding and the insoluble LDPE binder were eliminated by thermal debinding in an argon-rich environment using a tube furnace (HTF-15/200–60). 3YSZ/bimodal SS 316L micro-parts that had been solvent-debound were heated; the temperature was raised from room temperature to 300 °C at a rate of 0.1 °C/min. The thermal debinding process was eventually finished by raising the temperature by 0.2 °C/min to 600 °C for 3 h. In order to determine the ultimate circumstances of the degradation of palm stearin and LDPE binders from the 3YSZ and bimodal SS 316L portions of the 3YSZ/bimodal SS 316L micro-specimen, TGA was applied to the 3YSZ and bimodal SS 316L micro-specimens that underwent solvent debinding at 70 °C and thermal debinding at 600 °C.

### 2.5. Sintering Process

In this study, a comparable argon atmosphere was used for the sintering process, which came after the thermal extraction step. The thermally debound 3YSZ/bimodal SS 316L micro-components were heated for 3 h at a rate of 10 °C/min, increasing from 600 °C to three distinct temperatures of 1250 °C, 1300 °C, and 1350 °C during the sintering process. The earlier studies by Meng et al. [33] and Song and Evans [34] were taken into consideration when choosing the sintering temperatures. There were three stages involved in the cooling of the 3YSZ/bimodal SS 316L micro-samples produced in the tube furnace. The sintered samples were cooled from sintering temperatures to 800 °C, 400 °C, and 30 °C at cooling rates of 1 °C/min, 0.5 °C/min, and 0.3 °C/min, respectively. Measuring the length differences of five samples before and after sintering allowed for the calculation of the linear shrinkage percentage of the bimodally configured specimens. The density of the sintered 3YSZ/bi-modal SS 316L micro-parts was determined using the Archimedes method on the basis of MPIF standard 42. The morphology of the bi-materials was examined using FESEM. XRD and energy dispersive X-ray elemental (EDX) analysis were carried out to evaluate the phase and elements present at the bi-material interface. Hardness testing was carried out using a HIGHWOOD hardness tester (HWMMT-X series), with a load of 1 N and a dwell time of 15 s to verify the hardness of the joining region and the 3YSZ and bimodal SS 316L portions of the sintered bi-materials on the polish surface. Hardness measurements were obtained at three distinct locations, separated by 1 mm, for the 3YSZ and bimodal SS 316L portions of the bi-materials.

## 3. Results and Discussion

### 3.1. Material Characterisation

**Figure 3:**
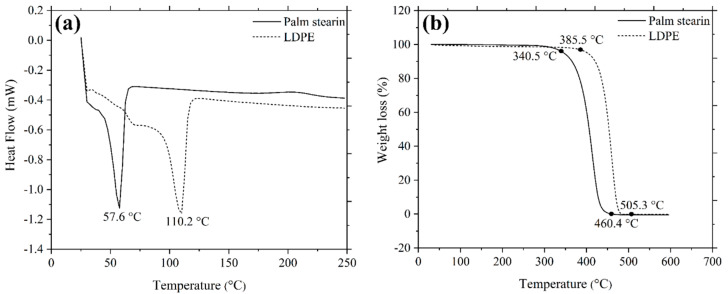
(**a**) DSC and (**b**) TGA curves of palm stearin and LDPE.

Figure 3a demonstrates the DSC curves of the palm stearin and LDPE binders. Palm stearin and LDPE began to melt at 57.6 °C and 110.2 °C, respectively. The mixing process was conducted at a temperature of 150 °C, which was higher than the melting point of LDPE, as it exhibited a higher melting point than palm stearin [35]. A TGA analysis was used to determine the degradation temperatures of palm stearin and LDPE; the findings are displayed in Figure 3b. According to Figure 3b, the decomposition of palm stearin started at 340.5 °C and terminated at 460.4 °C, while the decomposition of LDPE began at 385.5 °C and ended at 505.3 °C.

### 3.2. Preparation of Feedstocks

**Figure 4:**
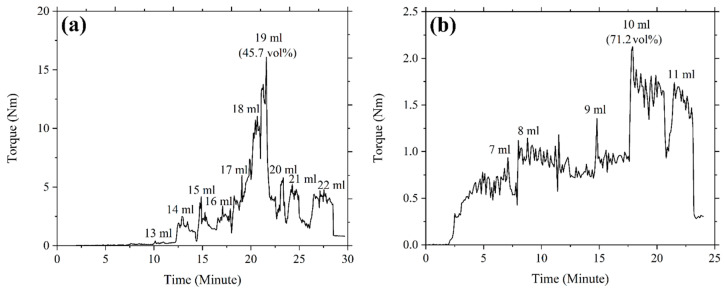
Torque variations over time of the (**a**) 3YSZ and (**b**) bimodal SS 316L powders during the addition of oil.

The loading of powder in a feedstock influences the physical and mechanical properties of the end product. The optimal solid loading should therefore be taken into consideration in feedstock formulation; this amount is typically established using the CPVC, which is the state whereby particles are packed as tightly as feasible despite the need for pressure from the outside and the binder fills every accessible space between the particles [14]. The use of optimal powder loading results in faultless sintered components with superior mechanical properties [36]. An exceedingly high feedstock viscosity frequently results from the use of critical powder loading, which complicates the micro-injection moulding process by preventing sufficient feedstock from flowing into the mould cavity. In this experiment, the CPVC of the 3YSZ and bimodal SS 316L powders was ascertained by gradually increasing the amount of oleic acid oil for altering the mixing torque. With the Brabender mixer, coupled with the torque rheometer, the torque was continually recorded. Figure 4 reveals that during the startup period, the torque increased marginally as a result of the oil being added to the mixture. After the oil was absorbed by the powder particle layer and the mixing torque stabilised, clusters started to develop. While oil was being incorporated into the mixture progressively, the torque generated by the expanding clusters increased significantly and continued to be unstable at the particular CPVC threshold. Solid structure dilatation, an enhancement of the interparticle distance, and a reduction in torque occurred when more oil was introduced into the mixture [31]. We used the following equation to find the CPVC percentage at the maximum torque:

(1) $$CPVC\left(\%\right)=\frac{V_{f}}{V_{f}+V_{0}}\times 100$$

where $V_{f}$ denotes the volume of powder and $V_{0}$ represents the volume of oleic acid oil. Based on Figure 4, the measured CPVC of the 3YSZ powder was 45.7 vol% when 19 mL of oleic acid was used, whereas the CPVC of the bimodal SS 316L was 71.2 vol% when 10 mL of oil was applied. Foudzi et al. [25] noticed a comparable CPVC of 45.3 vol% for the nano-sized 3YSZ powder. In contrast, Asmawi et al. [37] reported the CPVC value of 64.8 vol% for the micro-sized SS 316L powder, which was substantially lower than the CPVC value obtained in this experiment for the micro/nano bimodal SS 316L powder. This outcome revealed the significance of the bimodal configuration for increasing the powder content as the nanoparticles occupied the spaces between the microparticles in place of the binder (oil) [14,17]. Moreover, by comparing Figure 4a with Figure 4b, we found that the 3YSZ powder had a significantly lower CPVC value than the bimodal SS 316L powder. The reason for this could be that the 3YSZ nanopowder had a larger surface area, requiring a greater quantity of oil to thoroughly cover each particle [38]. Typically, the optimal powder content is outlined as a powder loading that is 2 vol% to 5 vol% less than the critical level [14]. As a result, in this research work, 44 vol% for 3YSZ and 69 vol% for bimodal SS 316L were determined to be the optimal powder loadings, on the basis of the critical powder loadings.

**Figure 5:**
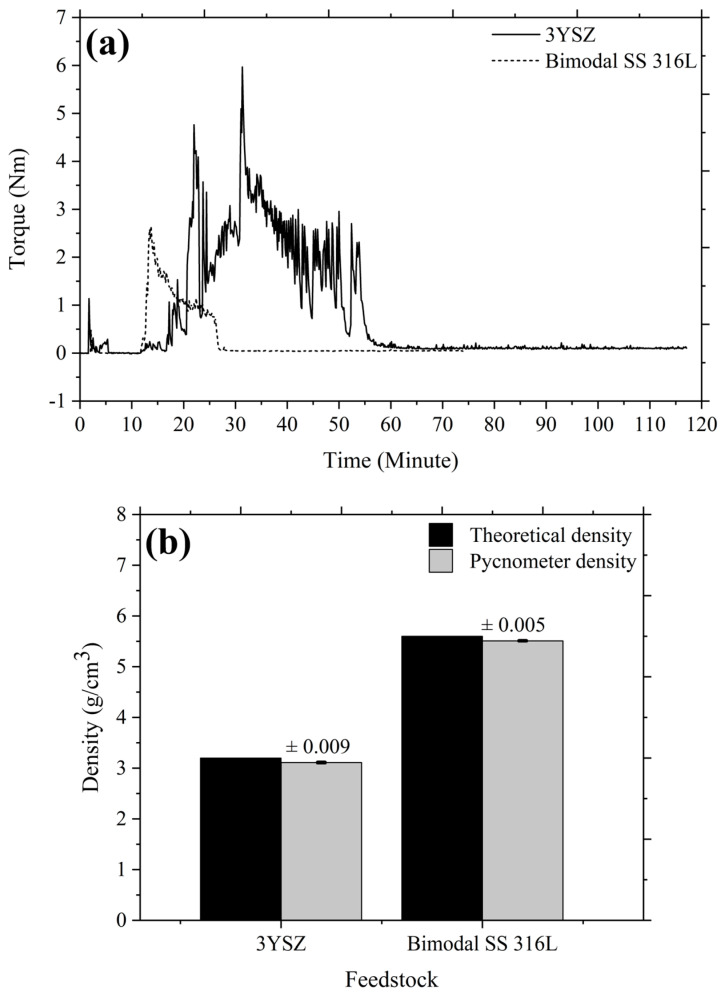
(**a**) Mixing curves of the 3YSZ and bimodal SS 316L feedstocks, and (**b**) theoretical and pycnometer densities of the 3YSZ and bimodal SS 316L feedstocks.

The mixing process in this investigation was performed with a twin-screw-blade mixer. The DSC and TGA studies, which are shown in Figure 3, were used to determine the mixing temperature. The mixing temperature of 150 °C was not only higher than the melting temperature of the LDPE binder but also lower than the initiation of the decomposition temperature of the palm stearin binder. The appropriate selection of the mixing temperature permitted the complete melting of the binders while also preventing their deterioration. Figure 5a illustrates the mixing curves of the 3YSZ and bimodal SS 316L feedstocks with optimal powder loadings of 44 vol% and 69 vol%, respectively. As shown in Figure 5a, the 3YSZ feedstock demonstrated substantially greater torque than the bimodal SS 316L feedstock, which could be related to the need for such torque to diminish the prevalence of agglomerated clusters in 3YSZ nanopowders. The 3YSZ and bimodal SS 316L feedstocks eventually reached a steady state condition following a sudden increase in the mixing torque. When a green body is being debound and sintered, an uneven density distribution can cause deformation and defects [39]. As a result, homogeneous feedstock is required to fabricate flawless components. In the current study, the homogeneity of the prepared feedstocks was validated based on the determination of the pycnometer density of five different measurements taken from a similar batch. Figure 5b shows a comparison of pycnometer densities with theoretical densities for 3YSZ and SS 316L feedstocks. The findings demonstrated a deviation of less than 0.5% from the average of the experimental density value, indicating that the powder–binder mixtures were sufficiently homogeneous [40]. Moreover, based on Figure 5b, the good agreement between theoretical and experimental values revealed that degradation of binders did not take place throughout the mixing process [40].

**Figure 6:**
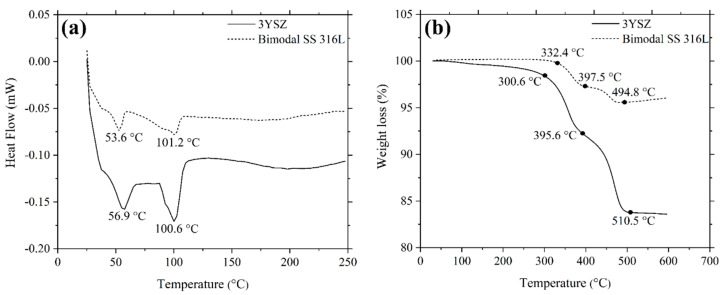
(**a**) DSC and (**b**) TGA curves of 3YSZ and bimodal SS 316L feedstocks.

Figure 6a exhibits the DSC curves for the 3YSZ and bimodal SS 316L feedstocks. The existence of two distinct types of binders was verified by the observation of two endothermic peaks in Figure 6a. The melting temperatures of the palm stearin binder were illustrated by the initial peaks for 3YSZ and bimodal SS 316L feedstocks, which appeared at 56.9 °C and 53.6 °C, respectively. In contrast, the second peaks, which became apparent at 100.6 °C and 101.2 °C, respectively, for the 3YSZ and bimodal SS 316L feedstocks, demonstrated the melting temperatures of the LDPE binder. A comparison of Figure 6a with Figure 3a revealed that the mixing process reduced the melting temperatures of the pure binders. This could be attributed to the binder absorption by the powder particles as well as the binder loss due to evaporation during the mixing process. The TGA results obtained for the feedstocks are exhibited in Figure 6b. As shown in Figure 6b, two phases of weight loss resulted in the decomposition of binders. While the first phase of binder decomposition in the case of the 3YSZ feedstock coincided with palm stearin and was completed between 300.6 °C and 395.6 °C, the LDPE binder was removed in the second phase at 510.5 °C. After the binders were completely removed, the mass that remained was 0.6% less than the mass of the 3YSZ powder used to prepare the 3YSZ feedstock. Similarly, for the bimodal SS 316L feedstock, the decomposition of palm stearin and the LDPE binders occurred within the temperature range of 332.4 °C–397.5 °C and 397.5 °C–494.8 °C, respectively. Following the expulsion of the binders, the remaining mass was 0.2% lower than the mass of the bimodal SS 316L powder utilised to produce the bimodal SS 316L feedstock. In this study, the debinding parameters were selected on the basis of the data obtained through TGA on 3YSZ and bimodal SS 316L feedstocks.

### 3.3. Rheology

**Figure 7:**
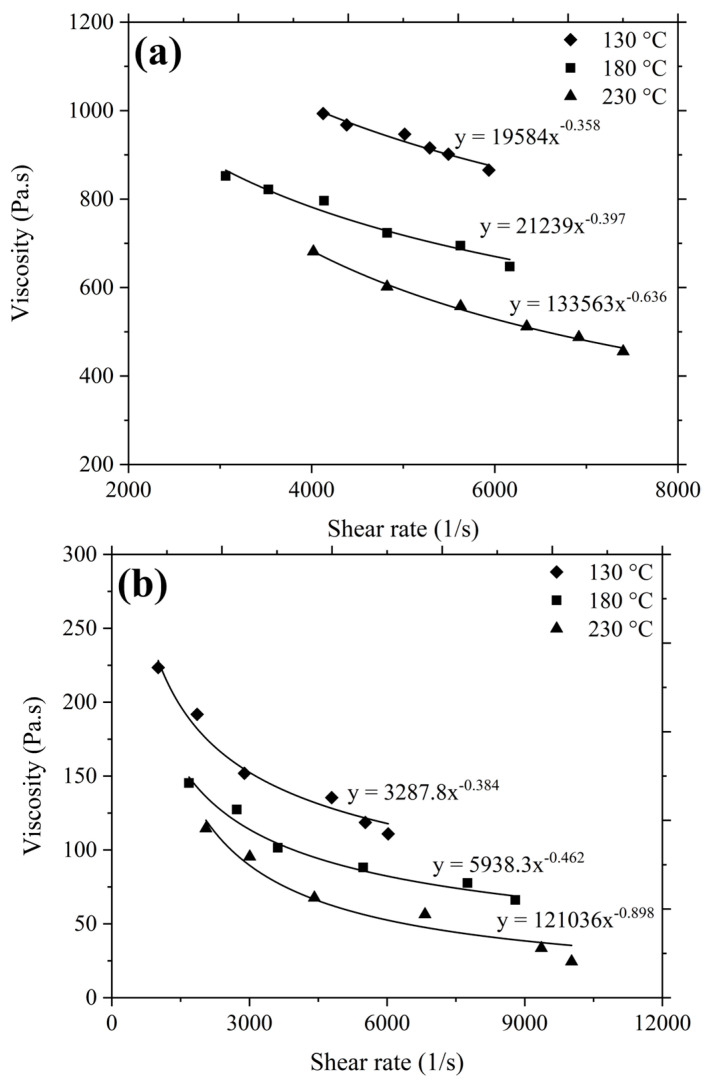
Variation of viscosity with shear rate for (**a**) 3YSZ and (**b**) bimodal SS 316L feedstocks.

The flow behaviour of the feedstock plays an essential role in determining the injectability of the mixture. The flowability evaluation of the 3YSZ and bimodal SS 316L feedstocks was conducted on the grounds of viscosity and shear rate sensitivity. Figure 7 exhibits the correlation between the viscosity and the shear rate of the feedstocks at 130 °C, 180 °C, and 230 °C. For both the feedstocks, the viscosity dropped when the shear rate increased. This outcome, referred to as shear thinning or pseudoplastic behaviour, confirmed that the feedstocks would adequately fill the mould cavity during the injection moulding stage [41]. As shown in Figure 7a, the range of viscosity for the 3YSZ feedstock was 865.5 Pa·s–993.4 Pa·s at 130 °C, which decreased to 455.6 Pa·s–681.4 Pa·s at 230 °C. Similarly, according to Figure 7b, the range of viscosity for the bimodal SS 316L feedstock was 110.9 Pa·s–223.4 Pa·s at 130 °C, which dropped to 24.4 Pa·s–114.5 Pa·s at 230 °C. This phenomenon was attributed to the molecules of the binders that exhibited lower attraction with increasing temperature [42]. According to the recommendations of investigators, the feedstock flow into the mould cavity was ensured by a shear rate range of 10^2^–10^5^ s^−1^ and a viscosity level of less than 1000 Pa·s [43]. The feedstocks prepared in this study had shear rates and viscosities that fell within the advised range.

The association between viscosity $\left(\eta \right)$ and shear rate $\left(\Upsilon \right)$ can be described by the following equation as the 3YSZ and bimodal SS 316L feedstocks exhibited pseudoplastic behaviour:

(2) $$\eta =K\Upsilon^{n-1}$$

where K denotes the constant and n indicates the flow behaviour index. The shear sensitivity is typically represented by the value of n, and the pseudoplastic behaviour corresponds to n<1. Table 2 provides the calculated n values for the feedstocks according to Figure 7, revealing that the values of n dropped with increasing temperatures. As the temperature of a feedstock increases, the mobility of the powder particles increases, ultimately lowering the value of n [42,44,45]. A smaller value of n denotes a feedstock with greater shear sensitivity, i.e., more pseudoplastic behaviour, and this is considered to be highly preferable for injection moulding, as during moulding, the feedstock viscosity needs to rapidly decrease as the shear rate increases [41,46,47,48]. In particular, the high shear sensitivity is crucial for 2C-µPIM to produce micro-sized bi-materials. In the present investigation, the lowest n values for both the 3YSZ and bimodal SS 316L feedstocks were observed at 230 °C. Therefore, this temperature was deemed the best for the feedstocks and used as the melting temperature during the injection moulding stage.

### 3.4. Fabrication of Green Part

**Figure 8:**
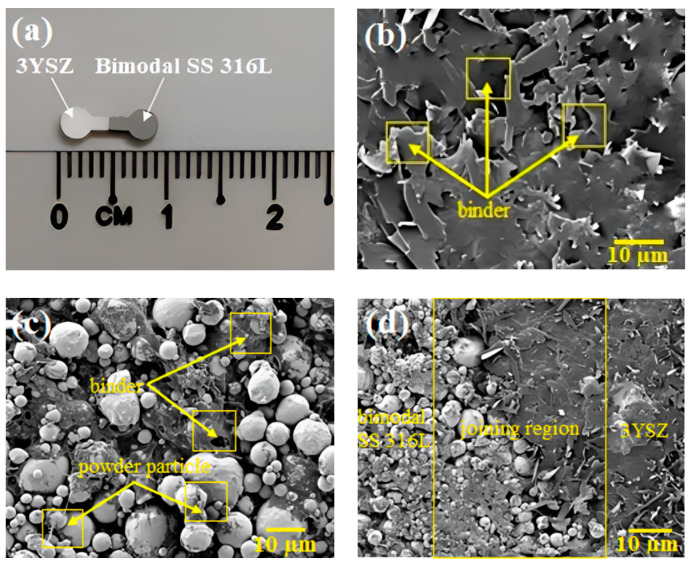
(**a**) Green 3YSZ/bimodal SS 316L micro-part, (**b**) FESEM image of the 3YSZ portion of green bi-material, (**c**) FESEM image of the bimodal SS 316L portion of green bi-material, and (**d**) FESEM image of the joining region of green bi-material.

During this experiment, no flowability of bimodal SS 316L feedstock was frequently demonstrated when the mould temperature was dropped below 100 °C. Based on earlier studies [14,42], the primary reason for this phenomenon is that the minuscule dimensions of the mould cavity cause the bimodal SS 316L feedstock to instantly cool during micro-injection moulding, restricting feedstock flowability at mould temperatures lower than 100 °C. This implied that the most significant factor influencing the filling of the mould cavity is the temperature of the mould. Figure 8a displays the green bi-material micro-part of 3YSZ and bimodal SS 316L that was produced when a mould temperature of 100 °C was used. A pressure of 12 bar was used for injection in this study as using a pressure greater than this resulted in flash defects in the components, while the use of a pressure lower than 8 bar restricted the amount of feedstock that could enter the mould cavity. Both of the feedstocks exhibiting pseudoplastic behaviour and low viscosity contributed to the production of the high-quality injected part. The FESEM images of the 3YSZ portion, the bimodal SS 316L portion, and the joining region of the green bi-material are exhibited in Figure 8b–d. These demonstrate that the powder particles of 3YSZ and bimodal SS 316L were adequately covered with the binders. As shown in Figure 8d, 3YSZ and bimodal SS 316L comprised the interface of the interlocked micro-components, and no gaps, cracks, or defects were detected there.

### 3.5. Elimination of Binders

**Figure 9:**
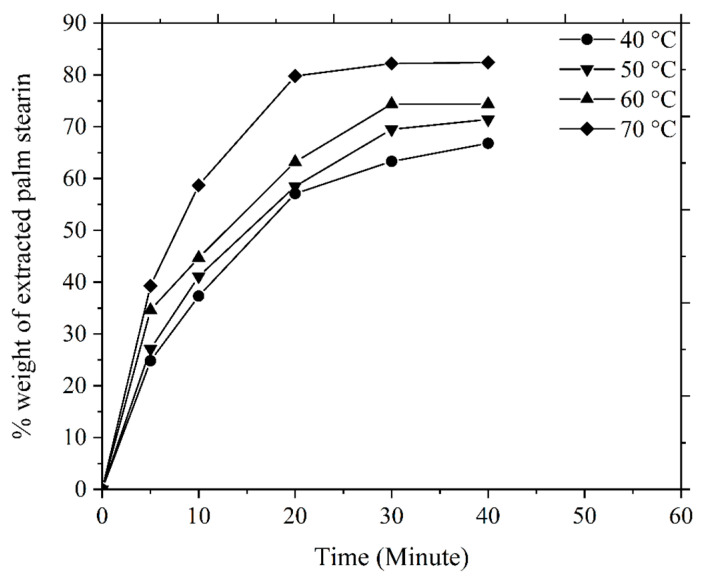
Mass loss of palm stearin in the green bi-materials at different times and temperatures.

The soluble binder of the multi-component binder system is typically extracted using the solvent debinding method, where the green components are immersed in a liquid solvent, such as acetone, heptane, hexane, or ethanol, and the procedure is carried out at a specific temperature. The diffusion mechanism serves as the basis for the elimination of the soluble binder [49]. In the current investigation, when the green 3YSZ/bimodal SS 316L micro-components were submerged in acetone, the solvent extraction process commenced with the diffusion of the molecules of the solvent into the palm stearin. This phenomenon resulted in the development of swollen gel. Palm stearin dissolved into the solution due to sufficiently strong interactions between it and the solvent that could overcome intermolecular forces [49,50]. The partial removal of the palm stearin binder in the bi-materials caused pore spaces to form, and as the debinding time and temperature increased, these pore spaces expanded to the interior of the bi-materials without impacting the insoluble LDPE binder. The amount of palm stearin removed from the green bi-material micro-components of 3YSZ and bimodal SS 316L with time at various temperatures is shown in Figure 9. Figure 9 exhibits that the mass loss of palm stearin increased with an increase in the solvent debinding temperature and time, which was consistent with the findings of earlier studies [30,51]. In the present research, increasing the temperature from 40 °C to 70 °C resulted in a significant increase in the rate of elimination of palm stearin from 66.8% to 82.4%. The elimination rate of palm stearin was found to be considerably high for the first 20 min at each temperature, and then it became slower or occasionally steady for the next 20 min. A key factor influencing the diffusion rate was temperature; as a result, a greater amount of binder removal occurred at higher temperatures because of the increased diffusion rate [52,53]. According to Basir et al. [36], the PIM and µPIM investigators most frequently utilised temperatures between 30 °C and 60 °C for solvent debinding. The chosen solvent debinding temperature for this investigation was 70 °C, as at this temperature, a significant proportion of palm stearin was eliminated. The use of solvent debinding temperatures above 70 °C typically increases the likelihood of crack formation and bi-material joining failure because of the rate at which palm stearin is removed and the softening of the backbone polymer.

**Figure 10:**
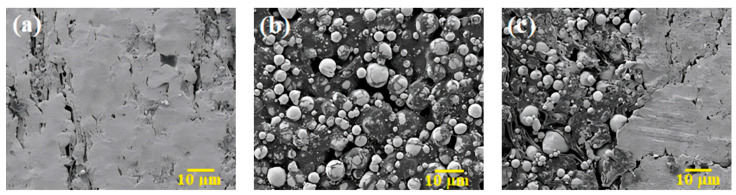
FESEM images of the 3YSZ/bimodal SS 316L micro-part following 40 min of solvent debinding at 70 °C: (**a**) 3YSZ portion, (**b**) bimodal SS 316L portion, and (**c**) joining region.

The FESEM images of the 3YSZ portion, the bimodal SS 316L portion, and the joining region of the 3YSZ/bimodal SS 316L micro-sized component after solvent debinding at 70 °C are presented in Figure 10. The overwhelming amount of palm stearin was eliminated from the joining, as well as the other portions of the bi-material, as shown in Figure 10, resulting from solvent debinding, in addition to the emergence of an open-pore configuration, which was crucial to eradicate the insoluble LDPE binder throughout the thermal debinding process. According to Figure 10c, the joining region of the bi-material was completely free from any types of defects that could cause complications in the subsequent processing stage.

**Figure 11:**
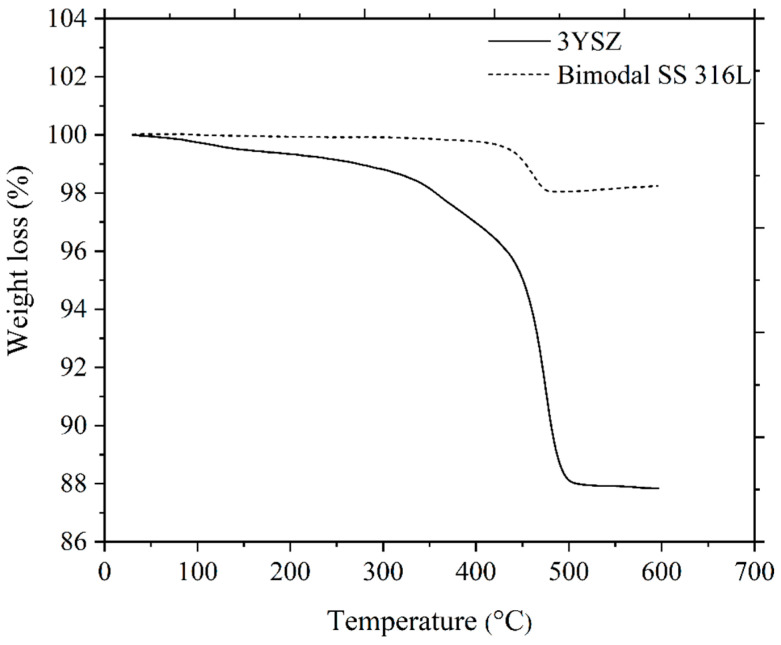
TGA of 3YSZ and bimodal SS 316L micro-components after solvent debinding.

As depicted in Figure 11, the single-material micro-sized components of 3YSZ and the bimodal SS 316L solvent debound in acetone at 70 °C for 40 min were subjected to TGA at temperatures between 30 °C and 600 °C. On the basis of the removal of 82.4% of the palm stearin from the 3YSZ/bimodal SS 316L micro-part and a comparison of Figure 11 with Figure 6b, we deduced that the 3YSZ portion of the bi-material contained the remaining 17.6% palm stearin within the decomposition temperature range of 300.6 °C–395.6 °C. Moreover, the removal of palm stearin from the bimodal SS 316L portion of the bi-material using solvent debinding was successful, as evidenced by the vanishing of the temperature range of 332.4 °C–397.5 °C. The decomposition temperature ranges for the 3YSZ and bimodal SS 316L feedstocks, which were 395.6 °C–510.5 °C and 397.5 °C–494.8 °C, respectively, as shown in Figure 6b, were still present in Figure 11. This clearly indicated that the 3YSZ and bimodal SS 316L portions of the of the solvent debound bi-material contained the LDPE. The thermal debinding procedure was therefore required to remove the LDPE and any remaining palm stearin binders.

**Figure 12:**
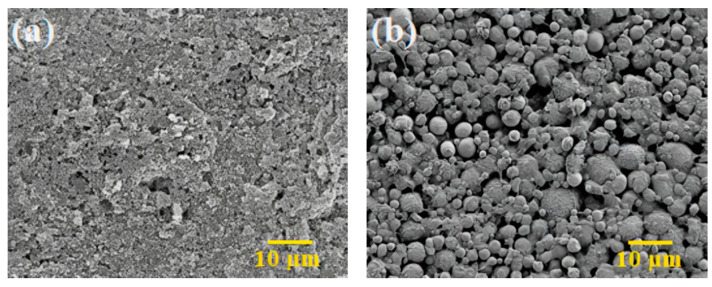
FESEM images of the 3YSZ/bimodal SS 316L micro-part after thermal debinding: (**a**) 3YSZ portion and (**b**) bimodal SS 316L portion.

**Figure 13:**
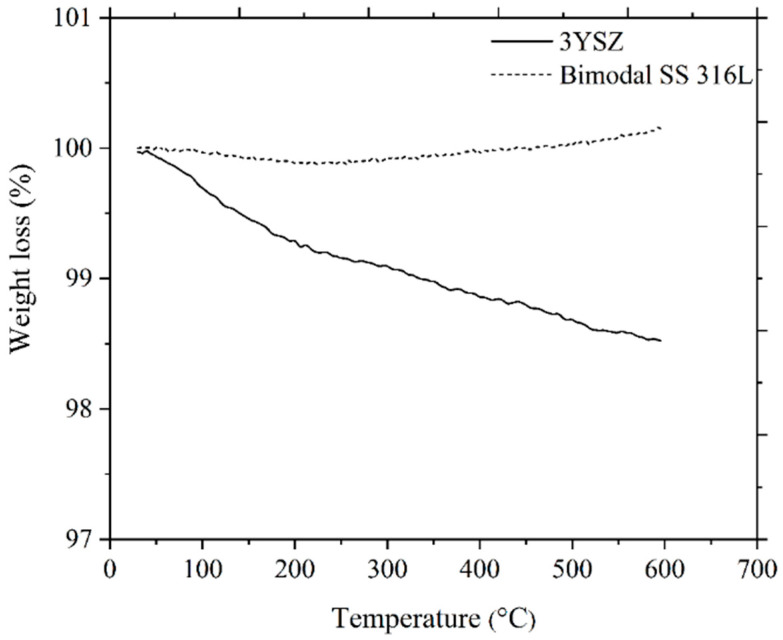
TGA of 3YSZ and bimodal SS 316L micro-components following solvent and thermal debinding.

The thermal degradation of the organic binders is the main objective of carrying out thermal debinding process, and the mechanism is based on the disintegration of polymers with the intention to produce light molecules that are evaporated out from the surface of the injection moulded components [49,54]. On the basis of the TGA assessment, as illustrated in Figure 6b, thermal debinding was carried out in this study. It was worthwhile to prevent the emergence of cracks and other flaws in the micro-components during the thermal debinding process by allowing the binders to gradually decompose across a broad spectrum of temperatures [55]. During the solvent debinding process, a considerable quantity of open porosities developed, which made it easier to remove the residual palm stearin and LDPE binders during the thermal debinding procedure [51]. In this study, the thermal debinding removed around 99% of the binder system, while an extremely small amount of leftover binder was removed during the sintering process. The FESEM images, as shown in Figure 12, revealed that the entire binder system had almost been eradicated from the 3YSZ and bimodal SS 316L portions of the bi-material that underwent thermal debinding. TGA was carried out on the 3YSZ and bimodal SS 316L micro-parts, which were subjected to the same solvent and thermal debinding parameters as the 3YSZ/bimodal SS 316L micro-parts. Figure 13 displays the obtained TGA curves. A comparative analysis between Figure 13 and Figure 11 revealed that the 3YSZ portion of the bi-material only held a very minimal quantity of LDPE after thermal debinding, whereas LDPE was entirely extracted from the bimodal SS 316L portion.

### 3.6. Sintered 3YSZ/Bimodal SS 316L Micro-Parts

**Figure 14:**
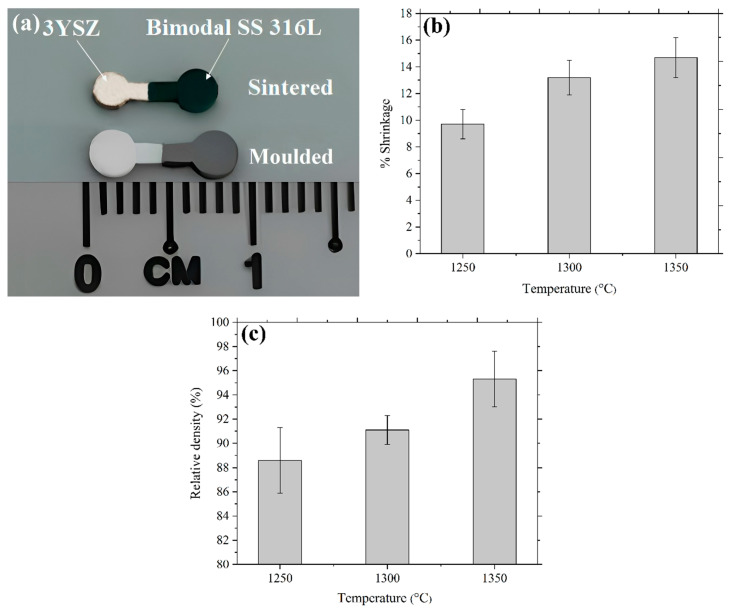
(**a**) Photograph of injection moulded and 3YSZ/bimodal SS 316L micro-part sintered at 1350 °C, (**b**) shrinkage in 3YSZ/bimodal SS 316L micro-parts at different sintering temperatures, and (**c**) relative density of 3YSZ/bimodal SS 316L micro-parts at different sintering temperatures.

Sintering eliminated the void space between the powder particles, which led to component shrinkage and densification. The 2C-µPIM-processed sintered 3YSZ/bimodal SS 316L micro-component exhibited a certain shrinkage, as illustrated in Figure 14a. According to Figure 14b, the shrinkage of the samples increased from 9.7% to 14.8% upon an increase in the sintering temperature from 1250 °C to 1350 °C. This outcome could be reconciled with the fact that at elevated temperatures, porosity lessened because of a diminution of the pore space between the powder particles. In the present study, enhancing the sintering temperature yielded an increased relative density. As demonstrated in Figure 14c, the relative density of 95.3% was achieved at 1350 °C. Usually, porosity declines and density improves when the sintering temperature increases [42]. According to Figure 14c, as the components sintered at 1250 °C and 1300 °C exhibited lower relative densities, it would be highly preferable to use 1350 °C as the sintering temperature for the fabrication of bi-materials.

**Figure 15:**
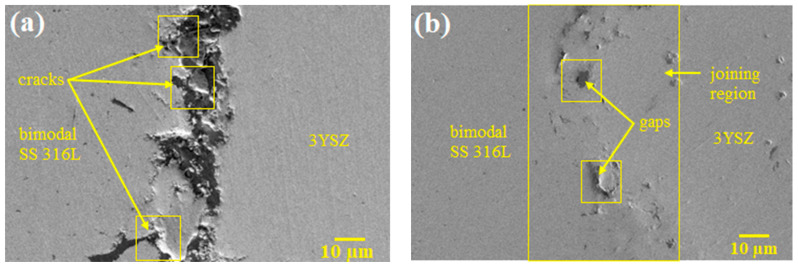
FESEM images of the 3YSZ/bimodal SS 316L micro-parts sintered at (**a**) 1300 °C and (**b**) 1350 °C.

**Figure 16:**
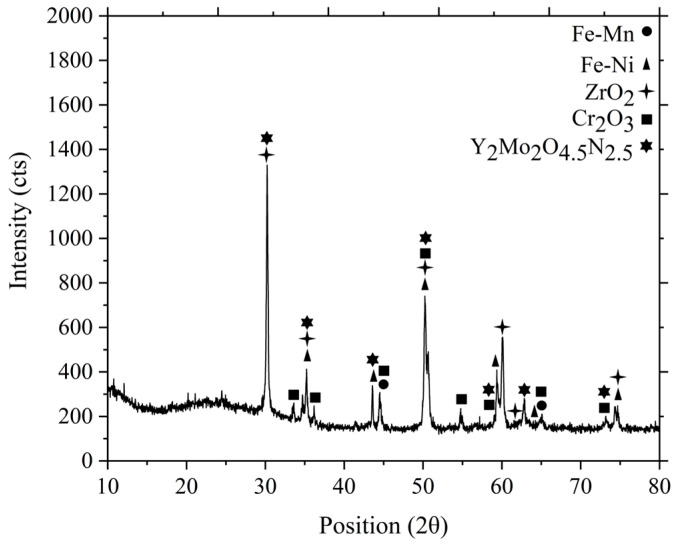
XRD analysis of the joining region of the sintered 3YSZ/bimodal SS 316L micro-part.

**Figure 17:**
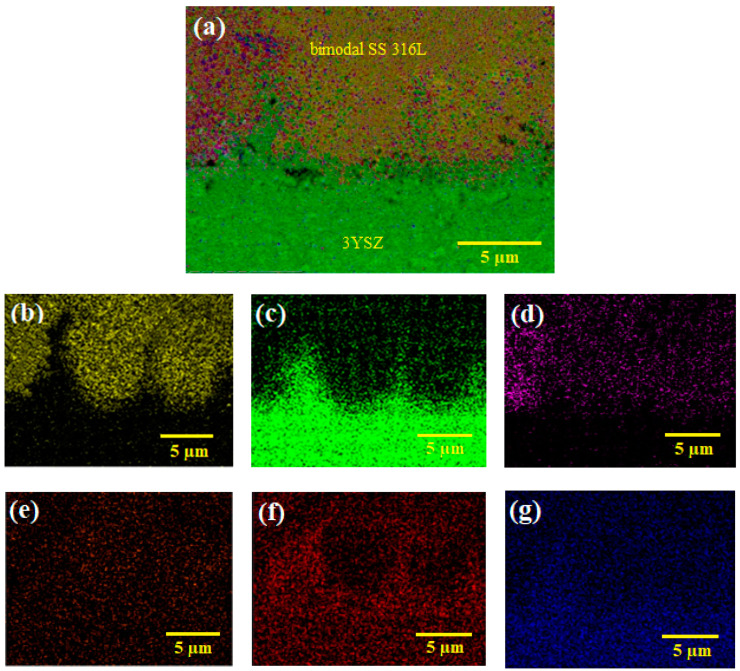
EDX mapping of the sintered 3YSZ/bimodal SS 316L micro-part: (**a**) layered image, (**b**) Fe map, (**c**) Zr map, (**d**) Cr map, (**e**) Ni map, (**f**) O map, and (**g**) Y map.

The FESEM images of the micro-sized bi-material sintered at 1300 °C and 1350 °C are displayed in Figure 15. As shown in Figure 15a, when sintered at 1300 °C, the partially bonded interface of 3YSZ and bimodal SS 316L demonstrated perceptible cracks. Increasing the sintering temperature to 1350 °C resulted in adequate bonding and the eradication of cracks in the bi-material, as illustrated in Figure 15b, despite the development of a few small gaps in the joining region. The XRD results of the joining region of the sintered bi-material are illustrated in Figure 16. It can be seen that several phases existed in that area. The diffraction peaks located at (2θ= 44° and 65°) and (2θ= 35°, 43°, 50°, 59°, 62°, and 74°) corresponded to the cubic and tetragonal structures of the Fe-Mn and Fe-Ni intermetallic phases, respectively. Fe-Mn possessed the lattice planes of (011, 020, and 121), and (100, 101, 110, 111, 102, and 112) crystal planes were related to the Fe-Ni phase. In addition, the presence of cubic ZrO_2_ and hexagonal Cr_2_O_3_ was confirmed as the diffraction peaks at (2θ= 30°, 35°, 50°, 60°, 62°, and 74°) and (2θ= 33°, 36°, 44°, 50°, 54°, 59°, 65°, and 73°) were detected. The former and later phases possessed the crystal planes of (111, 020, 022, 131, 222, 040) and (104, 110, 202, 204, 116, 212, 300, 119), respectively. Lastly, the diffraction peaks found at (30°, 35°, 43°, 50°, 59°, 62°, and 73°) were indicative of the cubic Y_2_Mo_2_O_4.5_N_2.5_ phase with (222, 040, 242, 044, 262, 444, and 080) lattice planes. Furthermore, no peaks associated with carbide phases were observed. It was reported that chromium had a high tendency to form chemical bonds with ceramics [56]. The phases formed in the studied samples demonstrated that chromium diffused to the ceramic/metal interface during co-sintering. In this process, the formation of an oxide layer and intermetallic phases may result in the absorption of stress discontinuities caused by mismatch strain during densification. The intense propensity for oxygen in 3YSZ acted as a key factor for the inter-diffusion of these components, resulting in bond formation. Figure 17 illustrates EDX mapping. The interface of the sintered 3YSZ/bimodal SS 316L micro-part contained Fe, Zr, Cr, Ni, O, and Y. Bonding between 3YSZ and bimodal SS 316L was eventually achieved through inter-diffusion of elements at the interface due to their greater affinity for the oxygen found in 3YSZ and the resulting generation of an oxide layer.

**Figure 18:**
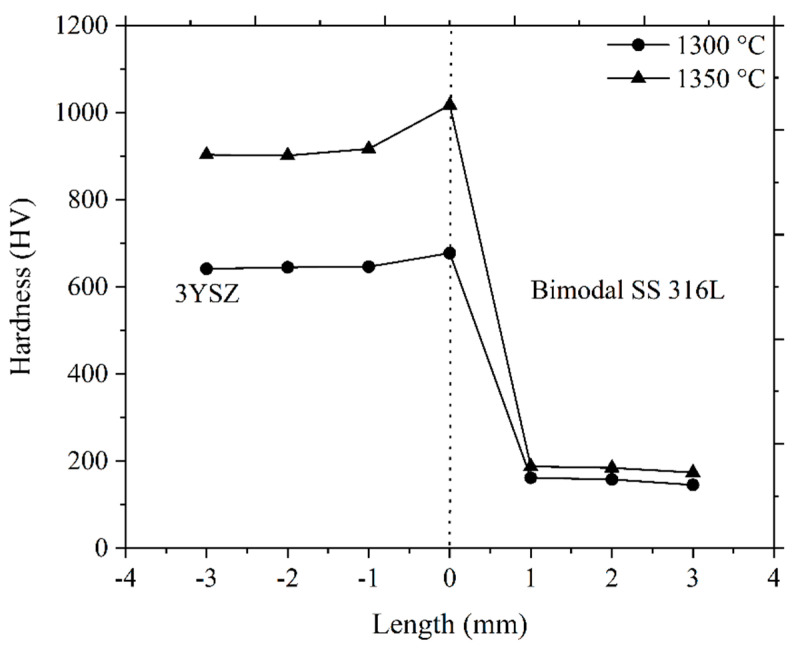
Hardness values of the bi-materials sintered at 1300 °C and 1350 °C.

The hardness of the sintered bi-materials was also measured (Figure 18). A dotted centre line in Figure 18 represents the joining region of 3YSZ and bimodal SS 316L materials. In this investigation, the hardness at various positions of the 3YSZ and bimodal SS 316L portions of the bi-materials increased as the sintering temperature increased. This was attributed to increased sintered density and grain refinement in the specimen [17]. Moreover, as shown in Figure 18, increasing the sintering temperature from 1300 °C to 1350 °C led to a significant increase in hardness along the joining region from 677.5 HV to 1017.4 HV. This result could be due to the elimination of cracks at a higher sintering temperature. Basically, the propensity of different material layers to shrink at varying rates causes the generation of bi-axial mismatch stresses at the interface during sintering, resulting in frequent cracks and gaps in the bonding region. Additionally, during the cooling phase of the co-sintering cycle, interface cracking may also result from variations in the coefficient of thermal expansion [57]. Furthermore, the hardness values achieved in this research work at the joining region at 1300 °C and 1350 °C are higher than the hardness values obtained from 3YSZ portions of the bi-materials. This could be attributed to the development of an oxide layer at the interface caused by the inter-diffusion of Fe, Zr, Cr, Ni, O, and Y elements during co-sintering. A follow-up investigation will examine the effects of the heating rate, holding time, and cooling rate during the sintering stage on the bonding as well as the physical and mechanical properties of the 3YSZ/bimodal SS 316L micro-parts.

## 4. Conclusions

This paper underscored the suitability of 3YSZ and micro/nano bimodal SS 316L feedstocks for the 2C-µPIM process to fabricate defect-free sintered bi-materials through the study of solvent and thermal debinding mechanisms. The following conclusions were drawn in light of the above-mentioned experimental results and discussions:

- The feedstocks demonstrated good homogeneity. The melting temperatures of the pure palm stearin and LDPE binders dropped after mixing separately with 3YSZ and bimodally configured SS 316L powders, inferring that the binder system was absorbed by the powders as well as evaporated during the mixing process. The rheological analysis of both feedstocks exhibited a pseudoplastic behaviour, with the lowest ranges of viscosity for 3YSZ and bimodal SS 316L at 230 °C being 455.6 Pa∙s–681.4 Pa∙s and 24.4 Pa∙s–114.5 Pa∙s, respectively, which was anticipated to be suitable for the 2C-µPIM process. According to a rheological evaluation, the green bi-material micro-parts of 3YSZ and bimodal SS 316L were fabricated at a mould temperature of 100 °C, as the use of a mould temperature lower than this would impede the flowability of the bimodal SS 316L feedstock into the mould cavity during injection moulding.
- While 66.8%–74.4% of the palm stearin binder was removed from green bi-materials during the solvent debinding process at temperatures ranging from 40 °C and 60 °C, the largest quantity (82.4%) was removed at 70 °C. The presence of the remaining 17.6% palm stearin in the 3YSZ portion and the LDPE binder in the 3YSZ and bimodal SS 316L portions of the bi-material solvent debound at 70 °C were revealed by a comparison between the TGA of the used feedstocks and the 3YSZ and bimodal SS 316L micro-components solvent debound at 70 °C. In this study, the thermal debinding procedure eliminated 99% of the binder system. Following thermal debinding, a TGA-based comparative study indicated that only a very little amount of LDPE persisted in the 3YSZ portion of the bi-material, whereas the bimodal SS 316L portion was totally free of LDPE.
- When sintered at 1350 °C, the solvent and thermal debound 3YSZ/bimodal SS 316L micro-parts showed a linear shrinkage of 14.8%; however, the shrinkage was substantially decreased at lower sintering temperatures. Increasing the sintering temperature from 1250 °C to 1350 °C resulted in an increase in the relative density from 88.6% to 95.3%. The microstructure of the bi-material sintered at 1350 °C demonstrated a reasonable bonding between 3YSZ and bimodal SS 316L. Finally, the hardness at the bonding region of the bi-materials increased drastically from 677.5 HV to 1017.4 HV when the sintering temperature was increased from 1300 °C to 1350 °C.

## Figures and Tables

**Table 1:** Micro-injection parameters to fabricate green 3YSZ/bimodal SS 316L micro-components.

Melt Temperature (°C)	Mould Temperature (°C)	Injection Pressure (bar)	Injection Time (s)
230	100	12	6

**Table 2:** Flow behaviour index at various temperatures for 3YSZ and bimodal SS 316L feedstocks.

Feedstocks	Temperature (°C)	Flow Behaviour Index (n)
3YSZ	130	0.642
	180	0.603
	230	0.364
Bimodal SS 316L	130	0.616
	180	0.538
	230	0.102

## Data Availability

Data that supports the results of this study are available from the first author.
